# Protocol for systematic review and meta-analysis of the evidence linking hippocampal neurogenesis to the effects of antidepressants on mood and behaviour

**DOI:** 10.1136/bmjos-2020-100077

**Published:** 2021-03-04

**Authors:** Juliana Aparecida Bolzan, Cilene Lino de Oliveira

**Affiliations:** 1 Departamento de Ciências Fisiológica, Universidade Federal de Santa Catarina, Florianópolis, Santa Catarina, Brazil; 2 Pós-Graduação em Farmacologia, Universidade Federal de Santa Catarina, Florianopolis, SC, Brazil

**Keywords:** hippocampus, neurogenesis, antidepressants, effect size, animal models

## Abstract

**Objective:**

Studies in rodents associated the deficits of adult hippocampal neurogenesis with behavioural anomalies which may be reversed by antidepressant treatments. A previous systematic review (SR) and meta-analysis (MA) indicated a hierarchy within the proneurogenic effects of different antidepressants in naive rodents. The present review aims to evaluate a more comprehensive sample of studies investigating the links between the effects of different antidepressants and adult hippocampal neurogenesis.

**Search strategy, screening annotation, data management:**

Protocols were planned following Preferred Reporting Items for Systematic Review and Meta-Analysis Protocols guidelines. Searches in Embase, Medline, Scopus and Web of Science followed by screening with inclusion/exclusion criteria will provide relevant publications. First SR will summarise the effects of antidepressants on adult hippocampal neurogenesis on different laboratory rodents. Second SR will summarise the correlations between neurogenic and behavioural effects of antidepressants while the third will focus on cause–effect relationships between them. If feasible, data will be analysed by pairwise or network random-effect or multivariate MA to determine the direction, magnitude, significance and heterogeneity (I^2^) of the estimated effect sizes on global or subgroup levels. Funnel plotting, Egger regression, ‘trim and fill’ will be used to estimate the risk of publication bias. Quality assessment of the included publications will be performed by applying adapted CAMARADES, Syrcles’ risk of bias or CINeMA tools.

**Reporting:**

Find a preliminary version of this protocol at the Open Science Framework (https://osf.io/gmsvd/). Data extraction has already started. Results shall be published in a peer-reviewed journal. Due to the continuous production in the field, the implementation of a ‘living SR’ is intended.

**Strengths and limitations of this study**

- A preliminary version of this protocol is registered at Open Science Framework.
- Search and screening cover an extensive range of publications.
- Two independent reviewers will perform data extraction.
- Quality of studies will be assessed with three different tools.
- Meta-analyses are planned to include indirect comparisons and non-dependent effect size.
- The protocol is not registered at PROSPERO because data extraction already began.
- Reviewers will be not blind to the bibliographic information of primary studies.
- A single reviewer performed search and screening, a second one double checked.
- Correlation between subgroups of studies may not be discarded.

## Introduction

The consumption of antidepressant medications used in psychiatric treatments doubled according to the Organisation for Economic Co-operation and Development between the years 2000 and 2015.^1^ Despite a large number of patients under treatment, the mechanisms underlying therapeutic effects of antidepressants remain elusive. Behavioural studies indicate that the therapeutic effects of antidepressants depend on the reversal of neurochemical deficiencies.^2^ For example, earlier studies revealed that most antidepressants inhibit the reuptake of monoamines, promoting high extracellular concentrations in the central nervous system.^3^ Moreover, antagonism of monoaminergic transmission provoked sudden decay of the effectiveness of antidepressants in patients.^4 5^ Although durable, the relationships between monoamines, depression and antidepressants may not explain all aspects of antidepressant therapy. For example, the delay between the onset of treatment and remission of symptoms or the existence of depressions resistant to prototype antidepressants suggests there are more for depression than monoamines.

Animal models allow for discoveries generating hypothesis and theories on depression and antidepressant efficacy beyond monoamines. Neurogenic theory of depression gained more attention in the 2000s when a series of papers reported that chronic antidepressant treatment increased the number of newborn neurons in the hippocampus of adult rodents^6–8^ suggesting a proneurogenic effect of the treatment. Stressful stimuli provoked hippocampal cell loss and behavioural sequelae prevented by chronic antidepressant treatment in rats^9^ indicating a positive correlation between proneurogenic and behavioural effects of the compounds. Furthermore, ablation of hippocampal neurogenesis reverted the effects of antidepressants on the behaviour of adult mice^7 8 10^ suggesting a cause–effect relationship between proneurogenic actions of antidepressants and their effects on behaviour. Altogether, these data engendered an explanation considering hippocampal neurogenesis as the underlying mechanism for the efficacy of antidepressants on treating mood and behavioural disorders.

Since the neurogenesis is a time-consuming process, the neurogenic theory offers a plausible explanation to delay between the onset of antidepressant treatment and the therapeutic effect. Neurogenic theory inspired a long series of studies aiming to understand details of the process which could provide a variety of new targets for the development of antidepressants. Over time the findings diverging of the earliest publications emerged indicating that some effects of antidepressants could be related to hippocampal neurogenesis while others would be unrelated to it.^11 12^ For example, the effects of chronic antidepressants on behavioural tests such as novelty suppressed feeding seem to correlate positively and depend on hippocampal neurogenesis.^7^ In contrast, the effects of antidepressants on the forced swimming test seem unrelated to it.^11^ A pioneer systematic review and meta-analysis (MA) of studies investigating effects of antidepressants in the hippocampus of adult laboratory rodents suggested that some compounds increased the neurogenesis while others did not.^13^ Depending on the type of the compound (fluoxetine, imipramine, venlafaxine, desvenlafaxine), laboratory species (rat or mice) and the type of molecular marker (BrdU, doublecortin, Ki-67), the magnitude of the effect size (ES) may vary from negligible to very large.^13^ The broad range of ES values indicates that actions of antidepressants may be more or less related or dependent on hippocampal neurogenesis.

To further explore the concept of the hierarchy of ES within different antidepressants, present work proposes to analyse the data of hippocampal neurogenesis with network MA. Network MA may enable indirect comparisons between ES.^14^ Additionally, the present protocol also plans to include studies of all times to perform a comprehensive systematic review and MA, which may help to overcome some of the limitations of the previous one.^13^ Although enough powered to evaluate ESs of different antidepressants on different species of laboratory rodents, boundaries of dates (5 years between 2013 and 2018) may have reduced the amount of data available to assess other sources of heterogeneity.^13^ Indeed, the information was insufficient to investigate the influence of relevant characteristics of the population such as sex, age and comorbidities (stressed, anxious and depressed phenotypes).^13^ Scarcity of relevant data also precluded the stratified analysis of the ESs of the antidepressants by doses, via of administration or efficacy in behavioural testing.^13^

Considering there is a variety of primary studies investigating the theme, it seems not feasible to obtain all relevant information with a single systematic review. In a pilot analysis, at least three categories of studies investigating the links between hippocampal neurogenesis and antidepressants in laboratory rodents were identified in the literature. For convenience, these categories were here named as 1-interventional, 2-correlational and 3-functional. Interventional category comprised those studies on the effects of the intervention (antidepressant) on the different markers of the hippocampal neurogenesis (neurogenic outcome), for example.^6–8^ The correlational category included the studies on the effects of the antidepressant (intervention) on a behavioural testing (outcome) and different phases of the hippocampal neurogenesis (outcome), for instance.^15 16^ Functional studies were those making ablation of hippocampal neurogenesis (intervention), cotreated or not with antidepressants, before behavioural testing (outcome), for example.^7^ Therefore, three different systematic reviews (SR) were planned to summarise interventional (SR 1), correlational (SR 2) and functional (SR 3) studies. In summary, SR 1 will further extend and update previous MA^13^ while SR 2 and SR 3 will explore newer aspects of the subject.

The ‘neurogenic outcome’, appraised in SR 1 and SR 2, often means the combination of different outcomes (measures of stemness, proliferation, survival, maturation of newborn neurons). Besides, every behavioural testing, evaluated in SR 2 and SR 3, should provide a different behavioural outcome (eg, immobility time in the forced swimming test, latency to touch the food in the novelty suppressed feeding, so on). Since random-effects model assumes independence of the ESs, but often information from the primary studies did not allow to discard correlations between them, current protocol plan to apply multivariate MA to take non-independence of data into account.^17^

## Methods and analysis

### Overview of the SRs and MA

**Figure 1:**
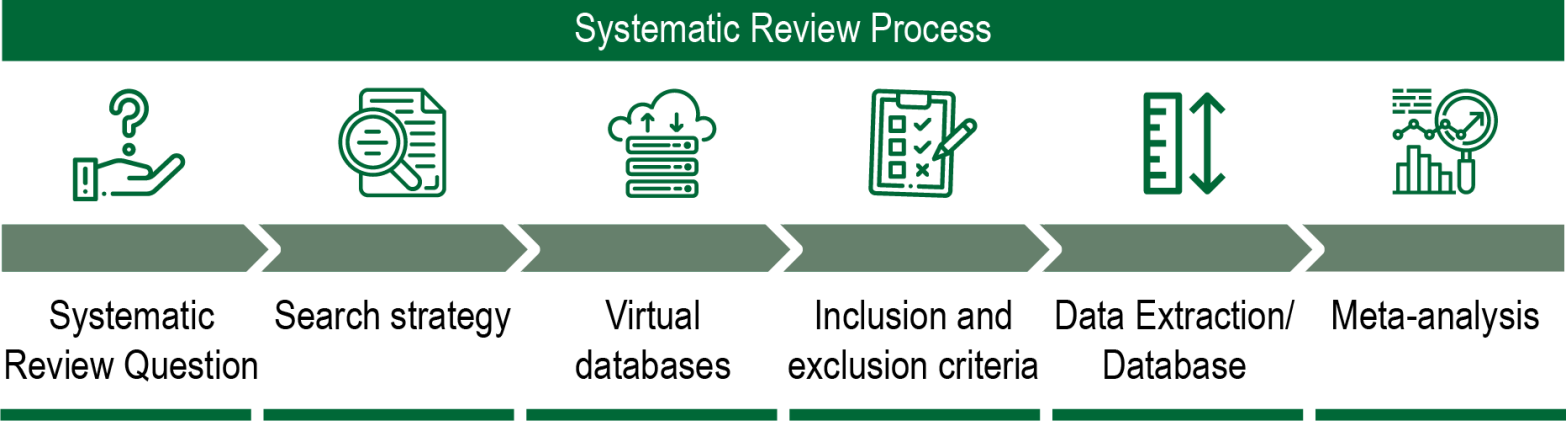
Timeline of a systematic review and meta-analysis. Icons in the illustration are available in inflaticon.com.

The protocol and the manuscript follow Preferred Reporting Items for Systematic Review and Meta-Analysis Protocols statement and Syrcle’s formulary. However, approval by the ethics committee and the Helsinki Declaration does not apply. The plan of every SRs (SR 1, SR 2, SR 3) included the following steps: (1) research question; (2) choice of search strategy; (3) choice of bibliographic databases; (4) choice of inclusion and exclusion criteria; (5) planning of data extraction and (6) planning of the MA (figure 1). SR 1, 2 and 3 aim to answer the following questions prepared using the PICO tool,^18^ respectively, (1) do different antidepressants increase hippocampal neurogenesis at different degrees in laboratory rodents? (2) are there correlations between behavioural and neurogenic effects of antidepressants in laboratory rodents? and (3) is there a causal link between behavioural and neurogenic effects of antidepressants in laboratory rodents?

In the three reviews, the population comprises laboratory rodents independent of species, strain, age, sex or comorbidities. Interventions and outcomes vary across SR, as follows. The SR 1 includes all studies investigating the effects of antidepressant treatment (intervention) on adult hippocampal neurogenesis (outcome), independent of the type of experimental design or association with behavioural testing. SR 2 will summarise the correlation between outcomes (hippocampal neurogenesis and behaviour). In those studies, the effects of antidepressant treatment (intervention) on adult hippocampal neurogenesis (outcome) and behaviour testing (outcome) are investigated in the same cohort of laboratory animals. Values of correlation will be extracted from primary studies, and when absent, calculated from the extracted data. SR 3 will condense those studies investigating the effects of ablating hippocampal neurogenesis (intervention) in laboratory rodent, with or without cotreatment with an antidepressant, evaluated in the selected behaviour testing (outcome).

**Figure 2:**
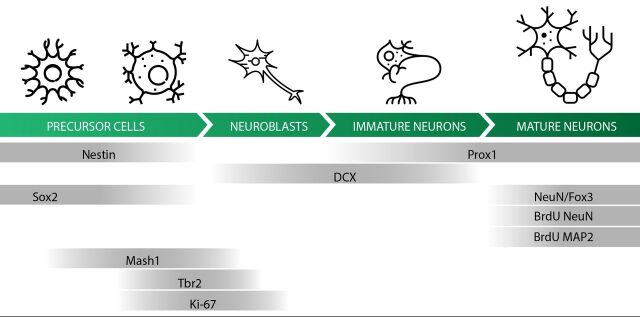
Adult hippocampal neurogenesis. Icons in the illustration are available in flaticon.com.

Neurogenesis will be assessed through the measures of different molecular markers of neurogenesis in the adult hippocampus (BrdU, Ki-67, DCX), arbitrarily chosen according to previously published SRMA.^13^ The different stages of neurogenesis (figure 2) will be estimated by quantification of the following markers on the dentate gyrus of the hippocampus: (1) Nestin (expressed in stem cells), (2) BrdU (incorporated by proliferating cell) or Ki-67 (expressed in proliferating cells, or other markers such as Tbr2, Mash 1, PCNA), (3) DCX (expressed in immature neurons), (4) BrdU and NeuN (expressed in mature neurons or other markers of mature neurons such as Map-2, Fox-3, Prox-1) colocalised (indicating mature neurons incorporated BrdU and matured in the adulthood). Measurements of the markers may be using stereological or non-stereological methods. The behavioural outcomes depend on the type of behavioural test reported (eg, time of immobility in forced swimming or tail-suspension test, latency to eat in the novelty suppressed feeding, so on). Data extract from each SR will be analysed separately. Analytical choices were made taken characteristics of the outcomes into account.

### Search strategy

The search strategies (see online supplemental table 1), (online supplemental table 2) and online supplemental table 3) at the Open Science Framework, access link: https://osf.io/tajhf/) were conceived after pilot studies using combinations of keywords from protocols previously published.^19–21^ Databases for the searches (“PubMed”, “Scopus”, “Embase”, “Web of Science”) were arbitrarily chosen considering their extensive collection of international publishers in the field of basic biomedicine and our institutional access. The keywords for SR 1 (online supplemental table 1) will be a combination of MeSH terms related to the primary outcome (hippocampal neurogenesis, #1–5 ‘neurogenesis’ and ‘hippocampus’ and (or ‘BrdU’ or ‘DCX’ or ‘Ki-67’) and intervention (antidepressants, #6). The keywords for the SR 2 will be terms related to behavioural outcomes (#7 online supplemental table 2) combined with the terms of SR 1 (online supplemental table 1). The search terms exclusive of the SR 2 were chosen by examining the publications obtained in SR 1. The keywords for the SR 3 will be terms related to the ablation of neurogenesis (#8, (online supplemental table 3) combined with the terms related to the behavioural outcomes (#7, (online supplemental table 2) and neurogenic outcomes (#1–5, (online supplemental table 1). Terms exclusive of SR 3 were chosen by examining the references similar to Santarelli *et al*.^7^ Publications returned of every search will be exported to a reference manager file of EndNote X7. Searches were performed by one reviewer (JAB) and will be double checked for a second reviewer.

### Screening processes

Screening of publications returned from the searches will occur in two rounds, the first one analysing title and abstract and second-round evaluating full text. The exclusion criteria are reviews, SRs, MAs, duplicates (all SR) and cotreatment (SR 1 and 2). The first round will exclude reviews, SRs, MAs, duplicates. The second round of screening will be required to identify cotreatments, exclusion criterion in SR 1 and 2, and apply inclusion criteria for all SRs.

**Figure 3:**
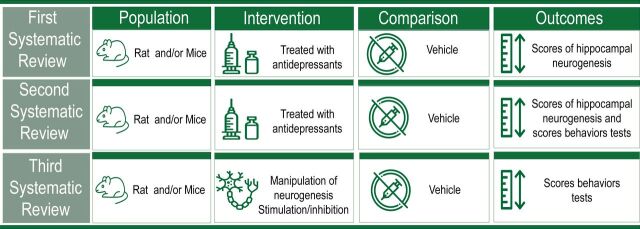
Types of primary studies included in every systematic review according to population, intervention, comparison and outcome. Icons in the illustration are available in flaticon.com.

All controlled studies will be included in SRs, regardless of randomisation, blinding or study design (figure 3). There will have no restriction on language or dates. Studies on adult rats or mice, regardless of sex, strain or stress background; studies with any route, dose and treatment schedule for drug administration; studies measuring any molecular markers in the dentate gyrus of the hippocampus (see figure 3) will be eligible. In these studies, interventions (antidepressants in SR 1 and 2; ablation of neurogenesis with or without co-treatment with an antidepressant, SR 3) will be compared with controls, that is, vehicle (SR 1 and 2) or sham ablation or the absence of the intervention (SR 3). Although studies with cotreatments will be excluded in SR 1 and 2, if the same study includes groups of animals treated with a single antidepressant and the respective control group, these groups will be included. One reviewer performed the screening process; a second reviewer will double check the screening. A first reviewer performed the screening process (analysing title and abstract and evaluating full text); a second reviewer will double check the screening. A third reviewer will solve discrepancies. Systematic reviewers will not be blinded to authors, date or journal of primary articles.

### Quality of study

Quality of every publication will be estimated by the use of an adapted version of CAMARADES checklist^22^ (box 1) and risk of bias (RoB) Syrcle tools^23^ which assess, respectively, general aspects of experimental design and more specific features of animal research. CINeMA tool will be used in the case of the implementation of the network MA.^24^

Box 1 **Adapted CAMARADES’ study quality checklist**

1. Peer-reviewed publication.
2. Studies following ARRIVE (or other) guidelines.
3. Declaration of compliance with animal testing regulations and legislation.
4. Declaration of interest.
5. Report of the breeding, husbandry conditions and actions to improve animal welfare of the experimental animals (eg, environmental enrichment).
6. Report of the species, lineage or other identifying characteristics of the experimental animals (eg, types of transgenes or knockouts) or phenotypes of interest (Stressed? Depressed? Anxious? Other?).
7. Report of the age, weight or stage of the experimental animals.
8. Report of the sex of the experimental animals.
9. Report of the methods of behavioural testing and acquisition of the behavioural outcomes.
10. Report of the methods to the detection of molecular markers, including the brand of antibodies and kits, and the acquisition of the molecular outcomes.

The quality of every study evaluated with adapted CAMARADES checklist will vary from score 0 (minimum) to 10 (maximum). Every study may receive a score when reporting information required in the checklist anywhere in the text (title, abstract or body of the text). This version of the CAMARADES checklist was adapted using items 6 and 9 of ARRIVE V.2.0 guidelines.^25^ A score at item 9 may be obtained by reporting (ARRIVE item 9): ‘(1) What was done, how it was done, and what was used? (2) When and how often?. (3) Where (including detail of any acclimatisation periods)? (4) Why (provide a rationale for procedures)’. A score at item 10 may be obtained by reporting (ARRIVE item 6): ‘Clearly define all outcome measures assessed (eg, cell death, molecular markers, or behavioural changes)’. The quality of every study evaluated using Syrcle’s RoB tool will be performed by answering the ten signalling questions with ‘yes’, ‘unclear’ or ‘no’, which will be considered a ‘low’, ‘unclear’ and ‘high’ RoB, respectively.^23^ The quality using CINeMA tool^24^ considers the following domains: (1) within-study bias, (2) reporting bias, (3) indirectness, (4) imprecision, (5) heterogeneity and (6) incoherence. To each domain, threes levels o judgement may be assigned: (1) no concerns, (2) some concerns or (3) major concerns. Judgements may be summarised to obtain four levels of confidence: (1) very low, (2) low, (3) moderate, or (4) high. Two independent reviewers will perform the quality assessment. A third reviewer will solve discrepancies.

### Outcome extraction

For a qualitative and quantitative summary of the included studies, bibliographic information and data on the experimental design, population, intervention, comparison, and the qualitative and quantitative aspects of the outcomes will be extracted from every publication (table 1). Qualitative data will be obtained from the text or tables. Quantitative data will be obtained from the text or tables of publications or using a digital ruler to extract them from the charts. When information is entirely unavailable in the publication, it may request directly by contact with the authors by e-mail of the corresponding author. In the absence of a response, within 30 days, the study will be excluded from the analysis. Two independent reviewers will extract data. A third reviewer will solve discrepancies. Every independent reviewer will input qualitative and quantitative data extracted from publications in an Excel sheet structured with a study per row and a variable per column. Agreement between reviewers will be assessed by using ‘comparing columns’ tool of the Excel (qualitative data) or by calculating Cohen Kappa index (quantitative data). Discrepancies above 20 %, that is, a minimum 80% of concordance considered substantial,^26^ will be conciliated by the third reviewer.

**Table 1:** Data extracted from each publication

Bibliographic information	Authorship, year, journal
Experimental design	See items in the adapted CAMARADES checklist (box 1) and RoB Syrcle tool^23^
Population	Species, strain, sex, age, the timing of euthanasia, phenotype of experimental rodents
Intervention	SR 1–3 (antidepressants): Type, mechanism of action, dose, via, number of injections per day. SR 3 (ablation of neurogenesis): the method employed to the ablation
Comparison	SR 1, 2: Type of vehicle. SR 3: the method employed to the sham ablation.
Outcome (qualitative)	SR 1, 2: Type of marker, antibodies used, the protocol for detection, protocols for BrdU injection (survival or proliferation), protocols of measuring (stereological or non-stereological). SR 2, 3: type, metrics and method of acquisition (manual or automatic) of the behavioural outcome.
Outcome (quantitative)	All SRs (per outcome, for experimental and control groups): mean, SD or SE, sample size (when available, effect sizes with 95% CI), p values or the values of F or t, and correlation indexes (SR 2).

RoB, risk of bias; SR, systematic review.

### Meta-analysis

Quantitative data extracted from publications (table 1) will be used in the MA. A similar protocol of the MA was previously published in the following studies.^13 27^ Data of each SR will be analysed independently. For every study included in each SR, an ES will be calculated by using a normalised mean difference and expressed Hedges’g with the upper and lower 95% confidence limits. For each SR, a global estimate of ES will include all laboratory animals, all antidepressants and all markers for neurogenesis. In the case of independent outcomes, random-effect models will be used for the MAs, and heterogeneity will be assessed by using the I^2^. Since the random-effects model assumes independence of the ES, when the primary studies do not allow to discard correlations between outcomes, multivariate MA will be performed. Multivariate meta-analysis may be used to estimate ES and heterogeneity taking non-independence of data into account.^17^

Stratified MAs into the subgroups (1) population, (2) intervention and (3) outcome are planned (table 2). Meta-regressions and, if necessary stratified, will be performed to evaluate doses of the antidepressants. For comparisons between ES of different antidepressants, network MAs are planned for data of each SR.^14^ Funnel Plotting, Egger regression, trim and fill, and p curve will be used to estimate the risk of publication bias. Calculations will be performed using R Studio.^28^

**Table 2:** Subgroup meta-analysis

Population	Species, strain, sex, age, phenotype, stress condition and behavioural testing.
Intervention	Type, mechanism of action, dose, via, number of injections per day.
Outcome	SR 1,2: Type of marker, antibodies used, the protocol for detection, protocols for BrdU injection (survival or proliferation), protocols of measuring (stereological or non-stereological). SR 2,3: type, metrics and method of acquisition (manual or automatic) of the behavioural outcome.

SR, systematic review.

### Prospects

The results of SR 1, SR 2 and SR 3 and the respective MA will be published independently in a peer-reviewed scientific journal. Implementation of a living SR (LSR) in this protocol may be useful to keep the SRs continuously updated. That is, relevant studies will be incorporated into the previously structured database of a formal SR as soon as they become available.^29 30^ Tools in bibliographic platforms such as PubMed offer periodical reports on an SR registered in the system. The LSR format is particularly important when a research field generates new data frequently. We expect that researchers interested in antidepressant research, especially those worried with mechanisms of action, may benefit from having updated information on this subject to plan new studies or make new synthesis to improve theoretical models in the field.
